# Imaging of the Unstable Shoulder

**DOI:** 10.2174/1874325001711010882

**Published:** 2017-08-31

**Authors:** Paolo Baudi, Manuela Rebuzzi, Giovanni Matino, Fabio Catani

**Affiliations:** 1 Department of Othopaedics and Traumatology Modena and Reggio Emilia University – Modena Hospital – Italy Via del Pozzo, 71 – 41124 Modena (Italy); 2 Department of Othopaedics and Traumatology Piacenza Hospital – Italy Via Taverna, 49 – 29121 Piacenza (Italy)

**Keywords:** Unstable shoulder, Shoulder imaging, Recurrent shoulder dislocation, Bone defect, PICO method, Hill-Sachs, Bankart lesion

## Abstract

**Background:**

Unstable shoulder can occur in different clinical scenarios with a broad spectrum of symptoms and presentations: first-time (or recurrent) traumatic acute shoulder anterior dislocation or chronic anterior instability after repeated dislocations.

Imaging in unstable shoulder is fundamental for choosing the right treatment preventing recurrence.

The goal of imaging depends on clinical scenario and patient characteristics.

**Method:**

Careful selection and evaluation of the imaging procedures is therefore essential to identify, characterize and quantify the lesions. Proper imaging in unstable shoulder cases is critical to the choice of treatment to prevent recurrence, and to plan surgical intervention.

**Results:**

In acute setting, radiographs have to roughly detect and characterize the bone defects present. At about 7 days, it is recommended to perform a MR to demonstrate lesions to labrum and/or ligaments and bone defects: in acute setting, the MRA is not necessary, because of effusion and hemarthrosis that behave as the contrast medium.

In recurrence, it is fundamental not only to detect lesions but characterize them for planning the treatment. The first study to do is the MRI (with a magnetic field of at least 1.5 Tesla), and if possible MRA, above all in younger patients. Then, on the basis of the pathologic findings as bipolar lesion or severity of bone defects, CT can be performed. PICO method on 2D or 3D CT is helpful if you need to study a glenoid bone loss, with the “en face view” of glenoid, while a 3D CT reconstruction with the humeral head “en face view” is the gold standard to assess an Hill-Sachs lesion.

**Conclusion:**

The clinical diagnoses of anterior shoulder instability can be different and acknowledgement of imaging findings is essential to guide the treatment choice.

Imaging features are quite different in chronic than in acute scenario. This requires appropriate indications of many different imaging techniques.

## 1 INTRODUCTION

An unstable shoulder can occur in different clinical scenarios with a broad spectrum of symptoms and presentations: from first-time traumatic acute anterior shoulder dislocation to chronic anterior instability after repeated dislocations. Dislocation can produce several types of injuries of varying severity: any of the labral-ligamentous complex, glenoid bone and/or proximal humeral epiphysis bone could be involved. The diagnosis can often be made on the basis of the medical history, but the diagnostic method and subsequent treatment choices are less obvious. Imaging findings range from undetectable capsular lesions to gross bone defects, with further complexity coming from the different anatomic variants of some structures.

Careful selection and evaluation of the imaging procedures is therefore essential to identify, characterize and quantify the lesions. Imaging in unstable shoulder cases is critical to the choice of treatment to prevent recurrence, and to plan surgical intervention; moreover, patient factors such as their profile in terms of biology and life-style, as well as any risk factors, are also to be taken into account in these choices.

The goal of imaging depends on the clinical scenario and patient characteristics; below anterior instability only will be discussed, for posterior and multidirectional instability imaging studies, see the specific chapters in this monography.

## 2 LABRUM-LIGAMENTS-CAPSULE COMPLEX INJURIES: DETECTION AND ASSESSMENT

Magnetic resonance imaging (MRI) is always considered the gold standard for the evaluation of soft-tissue injuries. The majority of surgeons use a 1.5 Tesla MRI machine. Magnetic resonance arthrography (MRA) in particular has shown high sensitivity (86-91%) and high specificity (86- 98%) for the capsulo-ligamentous complex, labral and cartilaginous structures [1]) Fig. (**1**). MRI is both less sensitive and specific, 44-100% and 66-95% respectively, for the detection and evaluation of glenoid labrum injuries. MRI is the gold standard for examination of rotator cuff and MRA for partial thickness cuff tears. Many studies have focused on the comparison between MRI and MRA in detection of glenoid labral injury; in 2012, a meta-analysis by Smith TO, *et al.* [2] examined sixty of these. The conclusion was that MRA showed a slightly greater diagnostic test accuracy over MRI in the detection of pan-labral lesions with a sensitivity of 88% and specificity of 93%, vs 76% and 87% respectively. But MRA is more costly, takes more time, is more invasive and exposes the patient to ionizing radiation if performed under fluoroscopic control, which in turn poses a low risk of adverse reactions. Moreover, if the data are separated taking into consideration only those relating to anterior labrum lesions, surprisingly MRI showed slightly superior accuracy than MRA [2]. But as far as SLAP and Bankart lesions are specifically concerned, MRA showed once again superior accuracy [2]. The glenoid labrum lesions are simply defined according to their location on the “glenoid clock”. A number of different terms are used to characterize supero anteroinferior labral injuries based on their patho-anatomy: Bankart lesion (complete detachment of the labro-ligamentous complex from the glenoid with disrupted periosteum) is the most frequent after traumatic anterior shoulder dislocation; Perthes lesion (non-displaced tear of the antero-inferior labrum from the glenoid, intact periosteal sleeve lifted off the scapular neck, inferior gleno-humeral ligament complex remains attached to periosteum); ALPSA (avulsed inferior gleno- humeral labroligamentous complex with medially displaced glenoid neck and intact periosteum); GLAD (a superficial tear of antero-inferior labrum with an adjacent articular cartilage injury); ALIPSA (avulsion of anterior band of inferior gleno-humeral ligament from glenoid origin but with intact labrum); HAGL (avulsion of the inferior gleno-humeral ligament from its humeral insertion); BHAGL (same as HAGL but with bony avulsion of the medial humerus); SLAP (rupture of varying severity of the superior labrum involving the long head of the biceps insertion). The best MRI sequence to study the labro-ligamentous complex is T1-weighted fat suppressed images in the transverse plane, with the exception of SLAP which is better detected in the coronal oblique plane.

Care should be taken to observe the anatomic variants, most importantly the middle glenohumeral ligament; the criteria for a ligamentous lesion evaluation are: rupture, thickness and degrees of intensity [3].

In addition to the use or lack of intra-articular contrast, and the power of the magnetic field, the image quality may vary considerably based on sequence types and the position of the upper extremity during the MRI investigation. The last consideration is true especially of the anterior band of IGHL complex studies, some authors have proposed the ABER position (ABduction and External Rotation) [2, 4]: by tensing this band, the anterior labrum is distracted with optimal penetration of the contrast material if tears are present, also in cases of joint effusions; but the greater accuracy of the ABER position remains not fully demonstrated [2, 5, 6]. MRA accuracy for antero-inferior labro-ligamentous structures is quite good both for acute and chronic lesions [7] and is better than MRI accuracy for many authors [8]; the more frequently-selected approach for intraarticular injection of diluted Gadolinium in MRA is the posterior one, using US-guide. But Applegate, *et al.* [9] suggested that a large joint effusion or haemarthrosis is produced in cases of acute traumatic dislocation: this fluid may be used as contrast material in conventional MRI Fig. (**2**). Therefore, MRA should be reserved for chronic instability scenarios.

Labrum-ligamentous-capsule complex injuries can be diagnosed also with arthro-CT, with a sensitivity of 93%, a positive predictive value of 93%, a negative predictive value of 33%, specificity of 33% and an overall accuracy of 87% [10]. Consequently, soft tissue injury is not sufficiently demonstrated with arthro-CT, which additionally, carries more side effects than arthro- MRI.

Ultrasound investigation is also indicated by some authors for the study of shoulder instability [11]. However, the majority of soft tissue injuries cannot be correctly evaluated. In contrast, US scan is a valuable diagnostic technique for rotator cuff tears, especially for incomplete ruptures [3].

## 2.1 Bone Defects: Identification and Quantification

Acute anterior shoulder subluxation/dislocation is often associated with either glenoid bone loss, humeral bone defect, or both [12, 13]. Recognition and assessment of the degree of glenoid and/or humeral bone loss in the preoperative stages play an increasingly important role in the planning of the appropriate treatment which will hopefully reduce the recurrence risk of unstable shoulder. In the last few years bone defects have received the same attention in the literature as soft tissue lesions [14-16].

Isolated glenoid defects are found in 22% of first-time anterior dislocation, and in up to 90% of chronic instability with repeated dislocations [12, 17, 18]. The prevalence of humeral head defects is variable in the literature, and appears to depend on the samples considered and the diagnostic imaging techniques employed. Bushnell, *et al.* [19] reported a frequency of 70% in acute anterior dislocations and 93-100% in recurrent instability. Ozaki, *et al.* [20] reported a prevalence of Hill- Sachs of 26.7% in primary subluxations and 73.3% in primary dislocations, in contrast the rate rises to 87.5% if patients with more than 1 episode are also considered. Kim, *et al.* [21] found Hill-Sachs lesions in 57.5% of acutely dislocated shoulders, while a prevalence of 94.5% was found in recurrent instability.

Up to 89% of recurrent shoulder dislocation presents gleno-humeral bone loss [17, 22].

### 2.2 X-Rays (Simple Radiographs)

Evaluation begins with high-quality plain radiographs, especially post reduction. Essential projections include: a true antero-posterior view in the scapular plane (Grashey view) with the arm in maximal internal-rotation, axillary view and Y view (less important). X-rays allow the identification also of associated fractures, which are more frequent in older patients, such as: tuberosities, surgical or anatomical humeral neck and coracoid (rare). Several other more specific projections could better detect and characterize the antero-inferior profile of the glenoid fossa: the West Point view (a particular kind of axillary view) and the Bernageau view.

Itoi, *et al.* [23] reported that the West Point view has shown a high correlation with computed tomography (CT) imaging in estimating glenoid bone loss.

Some authors have also reported the Bernageau view as a valid and accurate method for detecting antero-inferior glenoid bone loss: Edwards, *et al.* [24] reported that the Bernageau method can identify a glenoid rim defect in 79% of shoulders with anterior instability; Griffith, *et al.* [25, 26] found that images obtained with the Bernageau view are similar to axial images of 2-dimensional CT ; Pansard, *et al.* [27] state that glenoid bone defect measurement *via* the Bernageau profile view is a valid and reliable method that could decrease CT indications. To reduce the radioactive exposure of the Bernageau method, Sugaya has proposed a modified Bernageau method whereby patients lie on their axilla in the so called “TV watching position” [28].

Gerber, *et al.* [29] published that the true antero-posterior and Bernageau views detect bone loss of the antero-inferior glenoid profile with a sensitivity of 66% and specificity of 100%: however the limitation is the low reliability regarding the defect size; for detection of humeral head defects the Striker Notch view could be helpful, as it can better delineate their size and orientation; Bushnell, *et al.* [21] reported that only 57% of operative bony defects were detected on pre-operative radiographs.

Therefore CT imaging is required to obtain more accurate information each time a bone defect is identified on plain radiographs, or the clinical findings suggest their presence. CT scanning is the method of choice for studying glenoid bone loss [18, 25, 26, 30] and for humeral head defects, albeit with less sensitivity; however, there are few studies specifically examining CT scanning on Hill- Sachs lesions [20] compared to glenoid defects [23, 25, 30-33].

Following the detection of bone defects, the role of imaging shifts to their characterization and quantification for the purposes of treatment choice and planning. Depending on the severity of bone loss versus soft tissue damage, the surgeon should decide between the open or arthroscopic approaches.

### 2.3 Computed Tomography (CT)

CT scanning must answer some precise questions about bone defects: presence or absence, location, morphology and size.

For glenoid defects a location description can be expressed in relation to the glenoid clock face, typically they are situated on the antero-inferior rim, mainly anterior [25, 34]. Moreover, they can present morphologically with erosion (attritional bone loss) or fracture [18, 25, 26, 35]. Bigliani, *et al.* [36] coined the term bony-Bankart lesion and classified the glenoid bone defects into three types with increasing severity: the third type is bone loss: < 25% (3-A) and > 25% (3-B). Edwards, *et al.* [24] classified the glenoid defects into three types: bony-Bankart; loss of the antero-inferior angle with no visible fragment (cliff-sign) and bone loss (blunted angle). Griffith, *et al.* [26] also categorized into three types: the classic bony-Bankart lesion; “the straight pear” lesion, with a bone loss of 5-15% (the more frequent type in recurrent instability) and “the inverted pear” lesion, more severe as presenting with a bone loss > 20%. There is no consensus on the critical value for defining as severe or at high risk of recurrence after surgical repair without bone grafting, but most authors agree that the limit is about 20-25% of the total glenoid surface [16, 23, 30, 36-39].

The relationship between glenoid bone defect and recurrence is known. Rowe, *et al.* [40] reported a recurrence rate of 6-67% depending on the type of bone defect; Boileau, *et al.* [41] found a 75% recurrence rate among patients with at least 25% glenoid bone width loss; Burkhart, *et al.* [37, 39] found a recurrence rate of 89% in patients with 25-27% loss of glenoid width (“inverted pear” glenoid). This exemplifies how the real problem is quantifying the glenoid bone loss. Several methods have been used in CT to calculate the defect, grouped into linear (Bankart length measurement [29], Ratio method [42]) and circular (Best-fit or True circle method [28], Superimposed circle method [30], PICO method [32]), so the bone loss is calculated in mm or mm2 and expressed as the percentage of length or area of bone loss versus the total glenoid fossa respectively. Some methods calculate based on 2D-CT data with or without MPR (multiplanar reconstruction), others based on 3D-CT; some methods have the limitation of using the contralateral shoulder (if intact) as the control, but do gain in accuracy. The glenoid is always studied on the “en face view” which shows it in its entirety, with the exception of the humeral head.

**Fig. (3):**
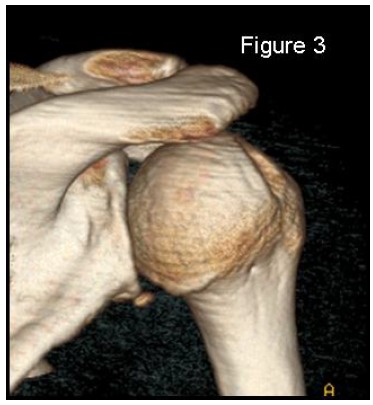
3D-CT is the gold standard to evaluate and quantify the humeral head bone defect. 54x59mm (150 x 150 DPI).

**Fig. (5):**
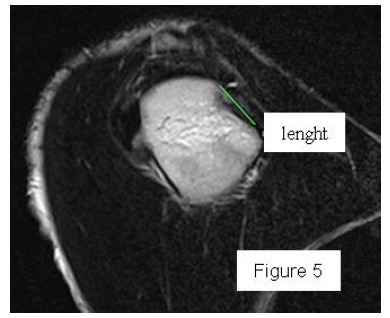
Measure of the lenght of Hill-Sachs lesion on CT sagittal view 55x46mm (150 x 150 DPI).

**Fig. (6):**
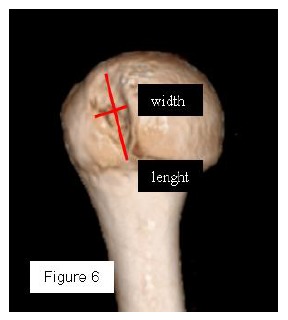
3D-CT humeral en face view with scapula elimination: the most accurate method to quantify the humeral bone defect (the minor axis is the width measure and the major axis is the lenght) 49x56mm (150 x 150 DPI).

**Fig. (7):**
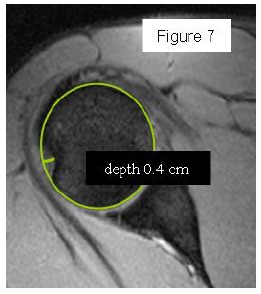
Humeral bone defect depth measure on CT axial view with the circle method. 44x49mm (150 x 150 DPI).

Most methods are based on the principle that the inferior part of the glenoid has a circular shape [43], attention must therefore be paid to positioning the centre of the circle. In an attempt to avoid bias in measurements, many authors have thought that 3D-CT methods could be more accurate, indeed Bois, *et al.* [17] comparing the accuracy of Glenoid Index [30], Ratio method [42] and PICO method [32] concluded that the PICO method is the best method albeit on 3D-CT. Magarelli, *et al.* [22] showed an agreement of 97% when calculating glenoid bone defects with the PICO method based on 2D and 3D-CT data. Therefore, to quantify glenoid bone defects any of these methods could be reproducible and accurate: the choice depends also on what kind of instrumentation is available. Above all, the crucial point is measurement accuracy: the glenoid has a width varying between 24-28 mm, so every 1.5-1.7 mm of missing bone correspond to 5% bone loss [39]. A defect of 6-8 mm results in bone loss of 20-25%, the critical value to opt for bone augmentation. For humeral head defects 3D-CT is the gold standard (Fig. **3**), especially for their characterization and to quantify the bone loss. The location and size are the most important features also for Hill-Sachs lesions [38, 44, 45]. The typical location is on the postero-superior portion of the humeral head, in contrast to the bare area which is postero-laterally located [34]. The orientation is, however, also important, indeed in 2007 Yamamoto, *et al.* [38] introduced the concept of “glenoid track” to describe the contact zone between the glenoid and the humeral head during combined abduction and external rotation movements. There is a risk of engagement (Figs. **4a**, **b** and **c**) with the glenoid rim if the head defect extends beyond the medial margin of said track. According to this theory, the depth and extent (width and length) are less important than location and orientation. If the long axis of the defect is parallel to the glenoid in abduction the external rotation engages its anterior rim, on the contrary if the long axis is diagonal in that arm position, it does not engage. In cases of intact glenoid the medial margin of the glenoid track is 18.4 ± 2.5 mm medial from footprint, and therefore a distance equivalent to 84 ± 14% of the glenoid width [38]. A recent study by Omori, *et al.* [46] has demonstrated that the width of the glenoid track at 60° of abduction was significantly greater than that with the arm at 90° or more. If a glenoid defect is concomitant, this could exacerbate the instability and increase the risk of a Hill-Sachs lesion to start engaging [47] and therefore reduce the area of the glenoid track, in contrast a larger Hill-Sachs could allow a smaller glenoid defect to cause engagement. Orientation was defined in relation to the deepest groove of the major axis of Hill-Sachs and the longitudinal axis of the humerus. The depth and length (and width though less importantly) are simply calculated both based on CT (Fig. **5**) and MRI data, ideally 3D-CT with elimination of the scapula (en face humeral view). The length of the major axis was defined as the length of the Hill-Sachs lesion, while the length of the minor axis as its width (Fig. **6**). On an axial view of the humeral head by CT or MRI (less accurate) a virtual circle that included the articular surface was drawn, and the depth of the lesion was defined as the longest length between the base of the Hill-Sachs lesion and the corresponding arc [20] (Fig. **7**).

These two parameters are considered in the prediction of clinical severity and risk of recurrence or engagement. Rowe, *et al.* [40] have graded lesions as mild (< 0.5 cm deep / < 2 cm long) up to severe (> 1 cm deep / > 4 cm long). Flatow, *et al.* [48] have quantified humeral bone loss as a percentage of involvement of the entire head area on 2D-CT and classified lesions into three groups: < 20%, 20- 40% and > 40%. Hill-Sachs defects > 40% are clinically significant, and this is recognized as the critical value requiring bone graft, while 20% is the threshold for remplissage procedure, even if the lesion is not engaged [49-51]; the critical measurements for Hill-Sachs lesions are: a depth more than 16% of the head diameter, an area more than 20-25% of the humeral articular surface, a volume greater than 250 mm3 (calculated on 3D-CT) [49, 52, 53]. Area and volume may be more convenient to compare the size of Hill-Sachs lesions, and the best images are those obtained from en face 3D-CT.

Di Giacomo, *et al.* [54] have recently proposed the concept of “on-track / off-track” lesions: if a Hill- Sachs lesion stays on the glenoid track (on-track lesion) the Bankart repair is recommended, if the lesion strays off-track the surgical procedure depends on the size of the glenoid defect. If it is smaller than 25%, remplissage combined with Bankart repair is recommended. But other authors have recently extended indications to perform remplissage even without substantial glenoid bone loss in large Hill-Sachs lesions and clinical instability [55].

The discovery of bipolar injury makes an accurate CT examination of both lesions even more imperative to avoid increased risk of recurrence: the glenoid and humeral head defects must be considered as two interrelated parts of the same problem.

MRI and MRA(conventional magnetic resonance and magnetic resonance arthrography).

Both MRI and MRA can be used for the identification and evaluation of bone defects (especially T2 weighted images), although non-displaced fractures can be difficult to detect after acute onset. However, it is in the quantification of defects that they show their greatest limit. Notwithstanding this, recent studies [43, 56-59] have demonstrated the validity and accuracy of both MRI and MRA in estimating glenoid bone defects compared with 2D and 3D-CT: MRA seems to be superior to MRI but its reliability is slightly lower than 3D-CT. Nonetheless, MRA is more sensitive and specific in presence of attritional bone loss compared to bony-Bankart investigations, and no study has reported MRI or MRA to be equal or superior to CT, especially 3D-CT, in the assessment of glenoid bone loss [45].

**Fig. (8):**
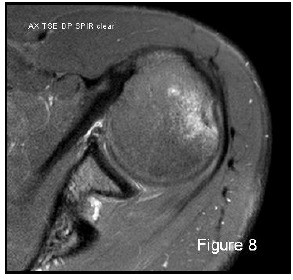
Altered signal intensity bone marrow oedema of the humeral head defect demonstrated on MRI. 61x58mm (150 x 150 DPI).

For humeral head defect detection the sensitivity of MRI is higher than that of CT and shows the greatest reproducibility [16]: this is due to the altered signal intensity of the bone marrow oedema demonstrated on both T1 and T2 weighted images, although fat-suppressed T2 weighted images are more sensitive (Figure 8). MRI shows high sensitivity (96.3%) and specificity (90.6%) for the presence of Hill-Sachs lesions (also chondral lesions), while MRA reaches a sensitivity of 100% even after surgical procedure. But the measurement of humeral defect size and the prediction of engagement or recurrent risk is an ambitious target for both MRI and MRA [60, 61]. Moreover, MRI can give false-positive results, misinterpreting erosions, small grooves or bare area for small Hill- Sachs lesions [34]; MRI importantly underestimates bone loss especially on the humeral side [62]. Furthermore, certain factors limit the large applicability of MRA in the quantification of bone loss: its high costs, invasiveness (relative), presence of ferromagnetic implants, possible allergic reactions and infections; on the other hand CT has the disadvantage of employing ionizing radiation.

### 2.4 Practical Imaging Based on Clinical Scenarios.

#### 2.4.1 Imaging in Acute Anterior Shoulder Dislocation (Primary or Recurrent)

In the acute setting patients arrive to the emergency room with anterior shoulder dislocation due to a traumatic event (more than 90% [59]). Two scenarios are possible: a first-time dislocation or an acute recurrent dislocation, often after a long period of good physical condition since the first or previous episode. Prior to dislocation reduction, X-rays are helpful only to show the direction of the dislocation, so an antero-posterior projection could be enough. Following reduction radiographs should include a true antero-posterior view in the scapular plane (Grashey view – Fig. (**9**) in maximal internal-rotation, an axillary view and an additional Stryker notch view to confirm the presence of Hill-Sachs lesions. Hill-Sachs lesions were more frequent and larger when the traumatic episode was dislocation rather than subluxation [20]. These projections have to roughly detect and characterize the bone defects present. During manipulation to reduce the dislocation, it is also possible to glean information about a possible engagement and which position is preferable for immobilization: to differentiate engaging lesions from non-engaging ones a functional assessment is required, as imaging is not useful.

At about 7 days, an MRI examination is recommended to show any lesions to the labrum and/or ligaments and bone defects: in the acute setting MRA is not necessary, because of effusion and hemarthrosis which behave similarly to the contrast medium in 97% of cases [63]. The inferior part of the labral-ligamentous complex could be more evident in the ABER position during MRI. Over thirty years old, the MRI or ultrasound examination are also important to reveal possible associated cuff tears. Following first-time traumatic anterior dislocation, IGL abnormalities were identified in 52% of patients on a conventional MRI scan within 7 days, compared to 12% by MRA 21-54 days later [64].

A first-time dislocation is not equal to an unstable shoulder, while even just a second or recurrent episodes exponentially greatly increase the risk of resulting instability; although the location and severity of structural injuries directly influence the risk of recurrence, patient age at acute dislocation also has a fundamental role.

#### 2.4.2 Imaging in Repeated Dislocations in Chronic Anterior Shoulder Instability

Imaging features are quite different in chronic compared to the acute scenario. In recurrent dislocations, it is essential not only to detect lesions, but to characterize them in order to plan the treatment. Repeated dislocations require progressively lesser degrees of force and provocation [65]. Patients therefore often arrive in the context of an orthopaedic studio visit.

In patients with recurrent dislocations, the prevalence of bone defects increases. Hill-Sachs lesions become larger with repeated dislocations, while recurrent subluxations do not influence the lesions [20]. The glenoid rim can become flattened and more deficient because of fractures and remodeling over time.

The first examination needed is a MRI and MRA especially in younger patients. Subsequently, on the basis of the pathologic findings, such as bipolar lesion or sever bone defects, CT can be performed.

**Fig. (11):**
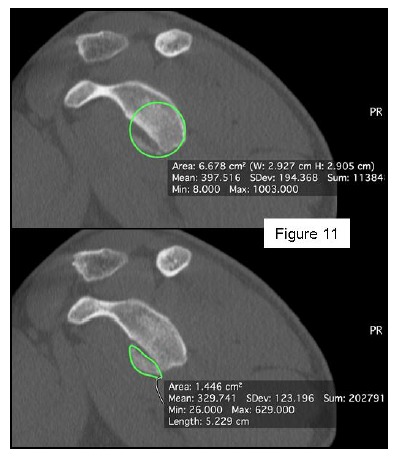
PICO method on 2D-CT en face view to quantify the glenoid bone defect area and percentage of bone loss. 87x101mm (150 x 150 DPI).

**Fig. (12):**
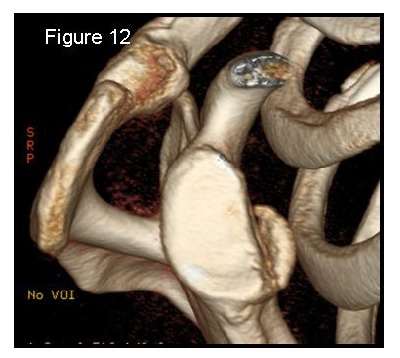
3D-CT reconstruction glenoid en face view to assess and quantify the glenoid bone loss. 62x56mm (150 x 150 DPI).

Patients presenting the most important risk factors such as recurrent dislocations, young age, long history of instability, strongly positive apprehension test at few degrees of abduction, bone defects visualized on radiographs [62] - should be carefully studied for the presence of bone loss to choose the best surgical technique for stabilization, open or arthroscopic. CT is currently the gold standard for the assessment of bone defects (Figs. **10a**, **b**, **c**), especially with 3D reconstruction images [66]. The main indications to perform a CT study are one or more of the risk factors listed above. The PICO method on 2D (Fig. **11**) or 3D CT is helpful if the “en face view” (Fig. **12**) of the glenoid is required to calculate the area and percentage of bone loss compared to the contralateral shoulder. A 3D CT reconstruction with the humeral head “en face view” is the gold standard to assess Hill-Sachs lesions.

## CONCLUSION

The clinical diagnosis of anterior shoulder instability can be difficult and acknowledgement of imaging findings is essential to guide the treatment choice. This requires appropriate indications of many different imaging techniques, taking into account the acute or chronic clinical scenario and the patient characteristics. The choice of imaging method must be targeted based on clinical suspicion of soft tissue and bone injuries, with correct timing and sequence.

In acute settings, the authors prefer X-ray imaging and MRI. In chronic scenarios, the authors recommend MRA, and if bone defects are found, a CT with 3D reconstruction with suppression of humeral head and scapula respectively to evaluate and better quantify the defects.

## Figures and Tables

**Fig. (1):**
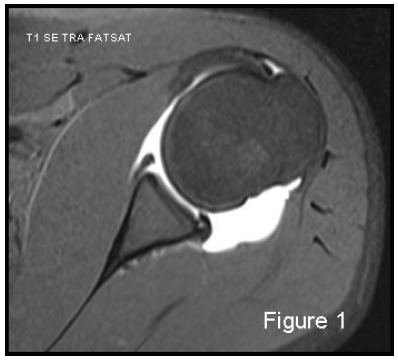
MRA: the best modality to visualize and assess the capsule-ligaments-labrum complex 59x53mm (150 x 150 DPI).

**Fig. (2):**
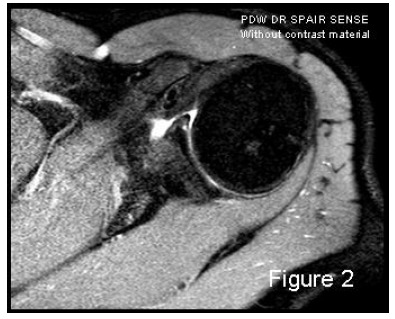
An example of MRI image that takes advantage of joint effusion as contrast material in acute scenario. 57x46mm (150 x 150 DPI).

**Fig. (4a):**
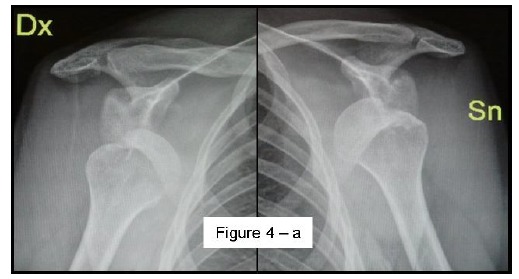
A case of bilateral engaging Hill-Sachs lesion on AP X-ray 108x58mm (150 x 150 DPI).

**Fig. (4b):**
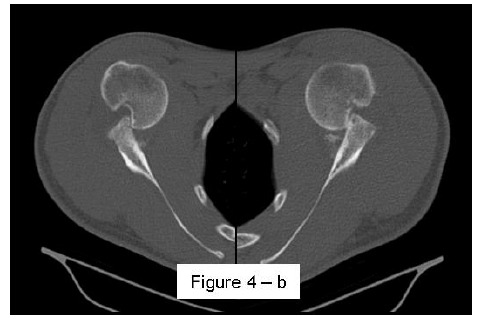
CT axial view with visualization of bilateral engaging wide and deep Hill-Sachs lesion 89x60mm (150 x 150 DPI).

**Fig. (4c):**
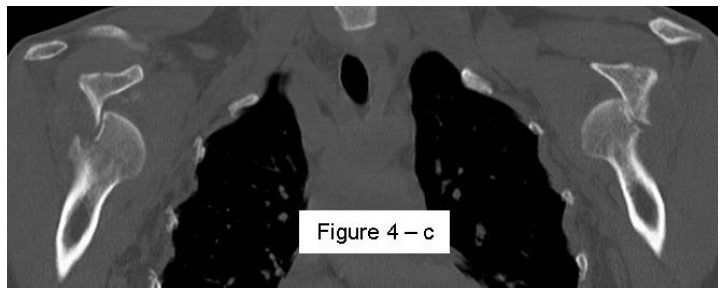
CT coronal view of the same case 111x44mm (150 x 150 DPI).

**Fig. (9):**
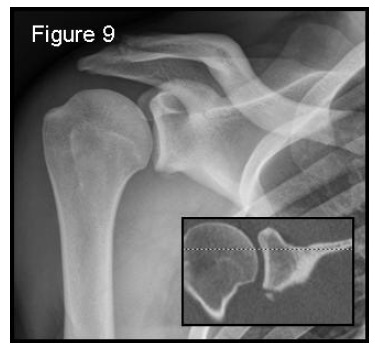
Grashey view: a true antero-posterior X-ray view in the scapular plane. 55x51mm (150 x 150 DPI).

**Fig. (10a):**
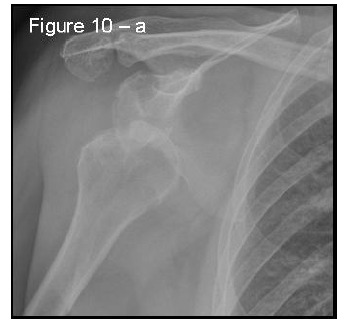
A case of bipolar bone defect on AP X-ray 57x55mm (150 x 150 DPI).

**Fig. (10b):**
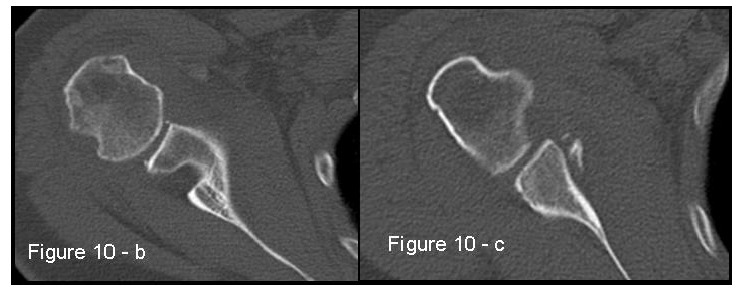
CT is the gold standard for the assessment of bipolar bone defect: **b**) Hill-Sachs axial view - **c**) antero-inferior glenoid margin defect axial view. 121x46mm (150 x 150 DPI).
